# The Report of the Social Science Association Committee on Quarantine

**Published:** 1862-01

**Authors:** 


### Art. V.—THE REPORT OF THE SOCIAL SCIENCE ASSOCIATION COMMITTEE ON QUARANTINE.

In 1858, the National Association for the promotion of Social Science, appointed a Committee to investigate the subject of Quarantine in all its bearings. The Committee was formed of the following gentlemen:—Dr. Babington, F.R.S., President of the Medico-Chirurgical and Epidemiological Society; Dr. Bryson, F.R.S., Inspector of Hospitals and Fleets; Sir James Clark, F.R.S., Physician to the Queen; Dr. Davy, F.R.S.L. and E., Inspector General of Army Hospitals; Dr. Farr, F.R.S., of the Registrar-General's Department; Dr. Gibson, C.B., the Director-General of the Army Medical Department; Sir John Liddell, M.D., F.R.S., Director-General of the Navy Medical Department; Sir I. Ranald Martin, C.B., F.R.S., Physician to the Secretary of State for India in Council; Dr. J. O. M'William, C.B., F.R.S., Medical Inspector of the Honourable Board of Customs; Dr. Gavin Milroy, F.R.C.P., late Medical Commissioner in the West Indies, and in the Crimea; Richard Owen, Esq., F.R.S., Member of the Institute of France, late President of the British Association of Science, &c.; Sir Wm. Pym, M.D., Superintendent-General of Quarantine; Dr. Southwood Smith, Member of the late General Board of Health; Spencer Wells, Esq., F.R.C.S., late Surgeon Civil Hospital at Smyrna and Renkioi; J. Wiblin, Esq., F.R.C.S., Medical Superintendent of Quarantine at Southampton; and three Members of Parliament (T. B. Horsfall, Esq., Thomas Bazley, Esq., and Walter Buchanan, Esq.), representing Liverpool, Manchester, and Glasgow.

In carrying out the investigation, the Committee framed a set of queries calculated to elicit information as to the actual existing condition, and the past results, of the practice of quarantine in foreign countries as well as in our own country and colonies. These queries were transmitted officially to all British Consuls abroad, and to the Governors of all British colonies, by order of the Foreign and Colonial Secretaries of State. In answer to these questions, information was transmitted to the Committee from every quarter of the globe, a digest of which was published in two Parliamentary Papers, one on "Quarantine Practice," and one on "Quarantine Laws," printed last session. Upon this information the Committee have drawn up a report, which now lies before us, and which must command the earnest attention of all who are interested in the question of quarantine, not only from the importance to be attached to the opinions of a Committee so singularly well qualified to examine the subject, but also from the intrinsic and unprecedented nature of the Report.

The subject, indeed, has been investigated in a strictly practical manner, having reference solely to the ascertained results of actual experience in all parts of the world; and not in respect of any one disease, but of all diseases which have hitherto been deemed liable to be imported from one country into another country. No similar inquiry has ever been previously attempted. All speculative and doctrinal discussions on contagion, &c., have been most judiciously avoided—indeed, they would have been quite unsuitable before such a Committee, and, as past experience has shown, could only have led to endless and most profitless disputes, both in and out of the Committee. The subject, moreover, has been examined, not merely as a doctor's question, but as a great social and international question affecting all classes, the merchant, the mariner, the statesman and the traveller, and the simple rule has evidently been followed of first collecting evidence as to the results of the system of quarantine hitherto pursued, and then drawing the obvious general conclusions from the evidence.

We propose to examine the conclusions of the Committee *seriatim;* adding such illustrations from the body of the Report as may serve to make the conclusions more obvious. Let us premise, however, after the fashion of the Report, that quarantine is by no means so simple an affair as many persons imagine. Not merely the detention for a limited time, and the purification of infected or suspected vessels, with their crews and cargoes, in consequence of the actual or the very recent existence of a dangerous contagious disease, either on board the vessel, or in the port from which she sailed, is meant or occasioned by it. This is, indeed, far from the reality. "In a large proportion of the cases where quarantine is still imposed in many countries, not only no sickness of any sort has existed in the vessel during the whole of the voyage, but no instance of the disease, on account of which she is subjected to quarantine on arrival, was known to have existed for a length of time in her port of departure. In the majority of cases, however, in which quarantine is imposed, its alleged necessity rests upon not a merely gratuitous apprehension, but upon the ascertained or the rumoured existence of a dangerous transmissible disease in the port or country from which the vessel has last come. All on board, indeed, may have been healthy during the voyage, and may be so on arrival, but the fact of the vessel having come from an infected or suspected locality, is held sufficient to require that she and everything on board should undergo a specified detention for the protection of the public health. The quarantine is directed against the *lieu de provenance*, or port of departure; and this is the reason why it involves all arrivals therefrom, without exception, whether sick or well; although when sickness has also occurred on board, the quarantine is usually more stringent than when the vessel has remained quite healthy during the voyage."

I. "*Great diversity and discrepancy* exist in *the system of quarantine* pursued in different countries; sometimes even in countries which are adjoining to each other, and under precisely similar conditions.

"Within the last eight or ten years, a great relaxation of the system has been made in some European countries, and likewise in certain colonies, while in other countries and colonies the system appears to be more vigorous than it was before. Much of the practice still in force is certainly uncalled for as regards the public health, and seems to be retained on fiscal rather than on sanitary grounds.

"Quarantine restrictions appear to have been sometimes resorted to from merely political motives, and to have been used as a pretext for the annoyance and detriment of other countries.

"All unnecessary interruptions to international intercourse, cause not only great personal inconvenience but serious commercial loss."

Illustrations of discrepancy and diversity of practice abound. At *Malta* no country is now subject to a permanent quarantine, all arrivals carrying a clear bill of health being admitted to free pratique; and at *Corfu,* "all arrivals from places where perfect health is generally enjoyed, and when furnished with clean bills of health, are freely admitted to pratique." In almost all other Christian ports in the Mediterranean a permanent quarantine is enforced throughout the year upon all arrivals from any port in the Ottoman dominions, including Turkey in Europe and Asia, Egypt and Barbary, *irrespective of the actual existence of the plague or other disease in the place of departure, or of any sickness on board the vessels during the voyage.*

The period of permanent quarantine (eight to ten days) in France and Sardinia is *inclusive* of the length of the voyage; in Spain and Portugal, and hitherto in the kingdom of the Two Sicilies it is *exclusive* of the length of the voyage, however protracted, and irrespective of the continued health of the crew.

The Governments of France and Sardinia reserve to themselves the power of modifying their quarantine codes and dispensing with any particular regulations when they see fit, and compatible with all due regard to the interest of the public health. In Spain and Portugal the quarantine codes are controlled and regulated in their operation by local boards of quarantine, or by a central board of quarantine, independently, in a great measure, of the general government of the country.

In France and Sardinia the quarantines have for some years been very mild in all cases. Even at Marseilles the system appears to be carried on with as little rigour as possible, and the mail steamers from Alexandria land their postage bags at once, however short the voyage may be. In the ports of Spain and Portugal the quarantine regulations are not only more stringent, but more regularly enforced; and in the Neapolitan ports the rigour of the quarantine, under the old regime, exceeded that of any other nation. Spain, for example, exacts a quarantine of observation on all ships arriving from ports (otherwise impeccable in quarantine laws) where strict quarantine measures, as in its own practice, are not duly enforced. The ports of Great Britain and the Northern United States consequently come within this category. Of 622 arrivals in the port of Alicante, during 1858, paying health dues, about one-third were from England; and at Malaga, while arrivals from England with a cargo of coals, and after a passage of from 25 to 40 or 50 days, are all quarantined for three days; arrivals from Egypt, with raw cotton, are admitted to pratique after eight or ten days' voyage! a quarantine version, as vexatious as ridiculous, of straining at a gnat and swallowing a camel.

The quarantines of Gibraltar are of necessity regulated by those of Spain, for if we did not impose nearly similar regulations Spain would close her communications with us, as happened in 1853. Malta is also at times compelled perforce, in order to quiet the apprehension of her neighbours, to adopt stringent quarantine measures, and also to put even a bar on Gibraltar; Gibraltar likewise putting a bar upon Malta! This happened in 1858, as the following examples from the Report of the extravagant, and utterly indefensible, folly of quarantine as practised in many Mediterranean ports. The illustration also shows in what manner the exaction by any one nation of stringent quarantine measures, it matters not how absurd and irrational these may be, reacts upon other nations and impedes the course of rational reform in quarantine laws and regulations.

"In the summer of 1858, on the first public announcement of the existence of the plague in the district of Bengazi, on the Barbary coast (the disease had been existing for months before its real nature was recognised), restrictive measures of extraordinary rigour were at once put in force throughout the whole of the Mediterranean and Black Seas, and in all the ports of Spain and Portugal, not only upon arrivals from the infected locality and other parts of the African coast, but upon contiguous countries and other places which might be supposed to be in direct communication with the seat of the fever, however healthy these places might continue to be.

"*Malta*, from its position and its trade with Tunis, &c., was exposed to especial suspicion; and accordingly the most stringent quarantine was enforced, even in various Ottoman ports, upon vessels arriving from or communicating with it; although, at the time, a quarantine of from 5 to 15 days was kept up by Malta upon all arrivals from the infected district in Barbary.

"*Gibraltar*, also, was similarly treated. A quarantine of 21 days' duration was imposed in the ports of Naples, Genoa, Portugal, &c., upon all vessels coming from or which had touched at Gibraltar. *Not that any disease existed there, or that the health of the 'Rock' was bad; but merely because it continued to hold communication with*

*Morocco, which was also at the time in a healthy state and quite free from any pestilential malady.*

"However unwillingly obliged to act in accordance with the rules and practice of other Mediterranean ports from the fear of retaliatory measures, Malta and Gibraltar had actually to put each other into quarantine; Malta, because Gibraltar had intercourse with Tangiers, &c., and Gibraltar, because Malta had intercourse with Tunis and Bengazi, *both British colonies being all the while in perfect health.*

"On the rumoured occurrence of a death from plague at Alexandria, and subsequently of a like occurrence at Beyrout during the autumn of that year (1858), a foul bill quarantine was established in all parts of the Mediterranean states against arrivals from these two places, and was rigorously maintained for many weeks, *when at length it was ascertained that there was no just ground for the rumours in question.*

"No case of disease was observed beyond the district around Bengazi where it first appeared."

In European ports quarantine is chiefly directed against plague, yellow fever, and cholera. The international conference of Paris on quarantine in 1851-52, recommended that the following periods of detention should be adopted in suspected, or alleged cases of those diseases in the ports of departure: *plague,* from 10 to 15 days; *yellow fever,* from 5 to 15; and *cholera,* from 3 to 5. But in the Neapolitan ports quarantine was enforced from 15 to 20 days in the cases of plague and yellow fever, and 10 to 15 days in the case of cholera. Similar lengthened periods have also recently been enforced in other Mediterranean ports, and in the oceanic ports of Portugal and her colonies. "In the autumn of 1857, the health authorities at *Madeira* refused to allow the landing of any passengers from the royal mail steamers, if any one had been taken on board at Lisbon, where the yellow fever then existed, although the vessel had remained quite free from sickness. The result was, that all the passengers bound for the island (chiefly invalids who intended wintering there) were obliged to go on to Brazil, and thus return to England as best they could." In the ports of Spain, in the case of yellow fever an average of 10 days' quarantine is exacted; at Marseilles from 3 to 7 days; at Bourdeaux the requirements are equally if not still more mild; at Malta, 5 days; while in the different ports of the American continent, and in the West Indies, the diversity and discrepancies of the regulations are so great, that no intelligent account could be given of the practice there. At *Carthagena* on the Spanish Main, at the island of St. Thomas and elsewhere, quarantine in suspected yellow fever has been formally abolished. At Demerara, in 1851 and 1854, forty days' quarantine was imposed upon ships coming from ports infected with cholera; and the regulations in the *Mauritius* require a detention of 20 days under the same circumstances. "In 1850, the municipal authorities of *Corfu* wished the governor to impose a 25 days' quarantine upon all arrivals from Trieste and Malta, and to order that all vessels from *Cephalonia* be rigorously prevented from approaching the shore, and *that some uninhabited rock be assigned to any of the inhabitants of Cephalonia* who sought to leave the island during the prevalence of cholera."

The diversity and discrepancies in the system of quarantine become even still more marked when we revert to the practice adopted in the Northern ports of Europe. There quarantine regulations have of late years become much less stringent than was heretofore the case. In 1854, Sweden imposed a most rigorous and vexatious quarantine on account of cholera; in 1855 she formally abolished quarantine on this account, as well as on account of yellow fever. "No vessel has been quarantined in Stockholm for several years past." At Hamburg formal quarantine regulations have been abolished; at Dantzig and Stettin, not a single vessel has been placed in quarantine during the last five years. At Amsterdam, and also in Great Britain generally, quarantine laws have become a dead letter.

How little much of the practice in force is called for, and even in some instances is regulated by considerations of the public health, may be gathered from the following examples: A steamer from Sierra Leone was recently refused pratique at *Teneriffe*, merely because smallpox was in that colony at the time of departure, "*although the disease was actually known to be in the island at the time.*" More than two-thirds of the vessels put into quarantine in Lisbon, in 1858, had clean bills of health. In the greater portion of these vessels the motive of quarantine was the ascertained, or *suspected*, existence of yellow fever or of cholera in the ports of departure. In most of the remaining cases, a death from some casual disease, or from accident during the voyage, was the assigned cause. "In one case the quarantine imposed was because the vessel from Gibraltar, with a cargo of leeches, had communicated with a steamer arrived from Alexandria then (erroneously) suspected of the plague." A vessel from Sunderland, and one from Hamburg, both with clean bills, were detained four and six days respectively in consequence of a death from *apoplexy* during the voyage!

The want of a duly formal bill of health, certified by the Spanish consul in the port of departure, has on several occasions been the motive of rigorous treatment. For example: In June, 1859, her Majesty's ship *Firebrand*, out twelve days direct from Plymouth, all well on board, had a quarantine of ten days imposed at Vigo, on this account. She sailed from Vigo in quarantine, leaving a mail from England, *which had been previously opened*

*on board and the letters pierced and dipped in vinegar before the authorities would receive them into their boat for landing.* Again: "In October, 1859, two English vessels from Glasgow, both having clean bills of health, the one bound for Oporto, and the other for Seville, put into Vigo from stress of weather. The first, having the Portuguese consul's certificate, was at once admitted to pratique; but the second was quarantined for three days, in consequence of the Spanish consul at Glasgow having annexed to his certificate this note: "The cholera has disappeared from this port, and from others comprised in an area of ninety miles, and all vessels are admitted to pratique, although coming from infected ports, provided there be no sickness on board."

The British consul at Alicante, in his replies to the queries of the Committee, doubtless laid hold of the true clue to the explanation of many of the inconsistencies and absurdities of quarantine, when he remarked, *apropos* of many detentions in the Spanish ports, that it would seem to have been imposed upon the ships "more for the purpose of obtaining the quarantine fees than as a sanitary precaution." In the Levant, if our experience does not mislead us, quarantine emoluments constitute a species of vested interest. In the Levant, moreover, it is generally believed that political motives are often at the bottom of measures taken in respect of quarantine. Thus, in 1850, the Piræus was suddenly put in quarantine, when Admiral Parker's squadron hove in sight. During the Crimean war, an attempt was made by the local authorities to put one of the principal Turkish ports in the Black Sea in quarantine, in order to annoy the English marine.

Some conception of the commercial loss inflicted by quarantine, may be gathered from the quarantine records at Malta, in the ten years 1845-54. The number of vessels quarantined during that period was 9,415, and the aggregate number of days spent by these vessels in quarantine no less than 47,430—*that is to say, an aggregate amount of time lost to commerce and subtracted from personal comfort or utility of very nearly* 130 *years in one port alone!* Singularly enough, notwithstanding the commercial aspect of the question, the different Chambers of Commerce in England have not co-operated with the Quarantine Committee in their inquiry, although requested to aid them by obtaining information as to the actual expenses of quarantine. Dr. Davy in a note to the Report, tells us that the expenses incurred at Malta in enforcing quarantine measures, at the time of the plague of 1813, amounted to the enormous sum of 232,536*l.* 13s. The sum of 1526*l.* was levied on eighty British vessels, during 1850, at Malaga, for quarantine expenses!

II. "The general *want of accurately detailed records* of the practice and results of quarantine from year to year, in different ports, prevents that full examination of the subject in its various bearings, which it is obviously the interest of all countries to possess.

"The *want also of faithful* official reports of the rise and progress of destructive epidemics in different countries, and more especially in British and other colonies, is for many reasons much to be regretted."

In no European port, we learn, is an annual report of the working and results of quarantine practice published, or even, so far as can be ascertained, prepared. In Canada, and also in certain of the States of the American Union, annual reports are made by the quarantine physicians and officers of health. Complete systematic reports were transmitted to the Committee from Sydney, (printed), and from the Mauritius (in manuscript).

The general absence of these reports is a curious comment on the *quasi*-scientific character of quarantine. The want of trustworthy *official* reports of destructive epidemics in different countries is a natural result of the system of quarantine as commonly practised.

III.—"The different forms of *bills of health* which are obligatory on vessels before leaving a port, and which profess to certify the state of public health in the *port of departure* and the surrounding country, are, as now given, obviously fallacious and often erroneous, and they can afford little or no reliable guarantee for the defence of the public health in the *port of arrival.*

"They are, in reality, passports to facilitate the admission of vessels on arrival, rather than trustworthy vouchers of a medical truth."

Plague had existed several months at Bengazi before the truth dawned upon the quarantine mind, and drove it to that hysterical outbreak of stringency which threw the whole trade of the Mediterranean into confusion. Plague did not exist at all in Alexandria in 1858, when quarantine went off into a paroxysm of absurd rigour upon a vague rumour. Yellow fever did not exist at *Para* at the beginning of 1860, although the Board of Health at Lisbon asserted that it did, declared the whole coast of Brazil to be infected in consequence, and subjected all arrivals from every port in that country to a foul-bill quarantine. Finally (not to multiply illustrations needlessly), yellow fever was quietly developing itself in Lisbon in 1856, while the Board of Health of that city, all unwitting that the disease was then at their thresholds, was watching it at the uttermost parts of the earth; and *typhus* has just struck down their Monarch, in his own palace; and yet they look abroad, and issue such notices as the following, quoted from the Shipping news of the *Times*, on the 3rd ult.:—"Lisbon, Nov. 26.—By an order from the Board of Health, dated yesterday, Loando is declared *suspected* of yellow fever, from the 1st of October." Truly, the philosophers of Laputa are not yet an extinct race!

IV.—"Even in those countries where quarantine is most rigorously observed, it is made to yield not only to the exigencies of war, but often also to the commands of superior governmental authorities in favour of particular arrivals, while other arrivals in a more healthy condition are subjected to detention."

At *Malaga*, on two very recent occasions, the quarantine regulations were set aside in favour of certain authorities and persons of rank who arrived there from places infected with cholera. A Royal Ordinance has also been issued, commanding that troops and military stores coming from infected places shall not be subject to quarantine. During the whole of the winter of 1859-60, while the cholera was raging in Africa, and it was well known that the Spanish army suffered severely from the distemper, having lost, according to public report, as many as 10,214 of its numbers; yet all vessels arriving from Ceuta or Tetuan with sick and wounded on board, were freely admitted to pratique in the ports of Spain. Exceptions are made in favour of men-of-war at Genoa, Naples, Malta, Gibraltar, in Turkey, &c."

V.—"The state of many existing lazarets is extremely faulty, and must inflict not only discomfort, but injury, on persons in health confined therein, while often no suitable accommodation is provided for the sick. To make use of a vessel placed in quarantine as a lazaret for the detention of her own crew and passengers, whether well or sick, is at variance with the teaching of modern medical science.

"The arrangements in most ports for providing *medical assistance to the sick on board* such vessels, appear to be generally unsatisfactory."

This conclusion is fully borne out by the facts recited in the Report; but we shall merely refer to those respecting the lazarets of Spain and the one at Lisbon, the two *ultra*-quarantine States.

Spain, it would appear, has only *two* regular lazaret establishments for the admission of foul arrivals: to wit, at Vigo and Port Mahon, such arrivals not being admissible into Cadiz, Barcelona, &c.; that is to say, that elsewhere the "foul arrival" passengers and crews are perforce confined to their ships during the period of quarantine. The Vigo lazaret is on a large scale, and is a "first-rate quarantine establishment," possessing all the arrangements for the reception of sick and suspected passengers, and for the landing and purification of cargoes.

The condition of the lazaret for the reception of passengers at

Lisbon is thus described by Dr. Donnet, of the Royal Naval Hospital, and forms a curious comment upon the quarantine rabidity of the General Board of Health of that city:—

"It (that is, the lazaret) consists of two buildings, separated from each other by a courtyard. The one is fitted up as a dormitory, and is badly furnished, badly ventilated, with the beds too close to each other, without either chimney or stove, and able to accommodate about fifty persons, although frequently it receives many more. . . . . The grounds around the lazaret are insufficient for exercise. There is no infirmary, no resident medical man or clergyman; no waterclosets, but in their stead, small night-stools placed in the dormitory, a curtain alone shutting out the occupant. The dormitory is frequently tenanted by both males and females."

Certain passengers from Southampton describing the miseries of this wretched and disgraceful place, in a remonstrance addressed to one of the daily papers, state, among other things, that:—

"Two gentlemen, their wives, a child, and maid-servant, were stowed away in a room without windows, and about nine feet square; five ladies found seclusion in a garret, while others, including three invalids, had to put up with the floor and benches of the dining-room, in some instances without a particle of bedding. No luggage was brought until the following morning; but, now that it is here, few have the opportunity of undressing for a change of clothing." The same authorities describe the urinals and a closet as "in a most loathsome and disgusting state, while the slopes beneath emit the most offensive stenches, and teem with rats and other vermin."

The floating lazaret is an old hulk at the quarantine ground, and is capable of receiving about seventy persons closely packed.

VI. "The experience of recent years appears to show that the *spread of a pestilential disease from persons or cargoes* undergoing quarantine in lazarets is scarcely known. Persons, however, going on board a foul and infected vessel on arrival, such as pilots, health-guards, custom-house officers, and others, have repeatedly been attacked with some dangerous disease soon afterwards.

"On several occasions, the disease for which quarantine was imposed has broken out on board, after the vessel has undergone the prescribed detention and been admitted to pratique."

The answers to the query (XI.) of the Committee, "Have any instances occurred in recent years of the spreading of a disease from persons, or from goods, undergoing quarantine, to other inmates of the lazaret, or to the officials of the establishment, or to the inhabitants of the nearest dwellings?" were almost uniformly in the negative. The few exceptions, real or apparent, in the mass of evidence transmitted to the Committee, were as follows:—

" *Lisbon.*—In July 1856, three deaths occurred among persons employed in the lazaret from cholera, which was then raging in the city, and where the disease seems to have been caught.

" The presumption that the first cases of yellow fever in 1857 were caused by the manipulation of infected luggage, which had been landed in the lazaret, is admitted to be simply conjectural. The fever was in the city in the previous year.

" *Malta.*—During the twenty-one years from 1819 to 1841, twelve vessels having, or having had, during the voyage cases of plague on board, were put in quarantine. In all, forty-six cases of the disease were treated in the lazaret, and of these cases twenty-two were fatal. The only instance in which the disease seems to have occurred among the employés of the quarantine establishment were in four health guards, two of whom had been put on board infected vessels, and the other two had been shut up in the lazaret to attend upon the sick. One of the latter died; the other three recovered.

" No instance has been known of ill effects from manipulating any description of goods received into the lazaret.

" *Southampton.*—Several suspicious cases have occurred.

" One man, an engineer, who landed from a West India steamer, was attacked with yellow fever some days afterwards, and died. A female in the same house was attacked with fever of apparently the same nature, but in a very mild form.

" Several other suspicious cases which put on the appearance of a modified typhus, it is supposed on account of the climate, occurred, but did not spread.

" In the case of Her Majesty's ship *Eclair*, which was ordered to move from the Motherbank to the quarantine station at Stangate Creek, a pilot embarked to take her in charge was taken ill with yellow fever three or four days after, and died.

" Two medical men were placed on board of her after she anchored at Stangate Creek; they were both attacked, but recovered.

" *Marseilles.*—In 1856 the persons in attendance on the sick in the lazaret, who were attacked with typhus fever, were five health-officers, two sisters of charity, one clerk, thirty-nine male nurses, and one soldier of the garrison; twelve of these persons died.

" *Grosse Isle, Canada.*—Almost every year, a certain number of the attendants whose duties bring them in contact with the sick, fall ill, and several have died in those years when fever (typhus) has prevailed. This was signally the case in the disastrous year of 1847, when many nurses, clergymen, and others fell victims in the discharge of their duties.

" *Baltimore.*—In 1852 an assistant physician and two nurses died from typhus fever, caught from sick emigrants. In 1857 one of the boatmen belonging to the hospital died of yellow fever, caught from contact with cotton landed from an infected ship.

" *New York.*—In 1856, many of the stevedores and others employed in unloading the sick vessels, were attacked with the (yellow) fever, and died. The disease spread to the shore, attacking first some dwellings on the beach near the hospital, and subsequently extending in different directions. Large quantities of refuse matter, decaying fruits, old bedding, &c., and all such materials as floated, were carried in directions and to localities which were subsequently the lurking places of the pestilence.

" 'There can be no doubt,' says the physician of the Marine Hospital, 'that the most active cause of the pestilence in this locality was from the accumulation of infected materials floated from the vessels in quarantine.'

" *Mauritius.*—None of the officials or police force at the lazarets ever caught either cholera or smallpox from the emigrants; but the wife of the lighthouse keeper at Flat Island died of the cholera during its prevalence there, and one of the crew of a steamer employed in carrying supplies to the lazaret on Flat Island, in 1856, took the disease, and died on board."

Dr. Lyons states, and his statement is confirmed by the inquiries of Dr. Donnet, R.N., that " the inspectors of the lazaret [at Lisbon], who had resided there for forty-two years, affirmed in the most positive manner, that there has never been a single person of those undergoing quarantine who was attacked with an epidemic disease." Four cases of cholera occurred in the lazaret of Madeira in the five years preceding its closure in 1858 : all recovered. Thirty-one cases of yellow fever took place in the Vigo lazaret in 1857, and three deaths from the same disease occurred in 1858 ; cases of cholera are recorded in the lazaret at Alexandria (two) in 1856; in the lazaret of Corfu (three) in 1850, when the disease prevailed in Cephalonia, and numerous cases of typhus in the lazaret of Marseilles in 1856—(soldiers probably from the typhus-stricken French army of the East.)

The questions involved in the communication of dangerous diseases to pilots, health-guards, and others going on board foul and infected ships, were treated by Dr. Milroy, in an article on the case of the Egyptian frigate at Liverpool, in the last number of this journal (p. 552).

The remaining conclusions of the Committee are as follows :—

" VII.—The classification of *cargoes* into susceptible or non-susceptible, retained in many of the Mediterranean and other Southern European ports, rests on a mere hypothesis unwarranted by experience; and the measures often adopted for the alleged purification and disinfection of ordinary cargoes in a sound and undecayed condition, are quite illusory.

"To fumigate, cut, or immerse letters, book-parcels, &c., is simply absurd.

"VIII.—It does not appear that those countries in which quarantine restrictions are most rigorous and most strictly enforced, have hitherto been more exempt from the visitations of the diseases against which quarantine is chiefly imposed, than other countries where the regulations are more simple and less burdensome.

"It is highly important that sound views respecting quarantine be held and acted on, as all unnecessary or erroneous measures of sanitary police not only cause annoyance or positive mischief, but serve to withdraw public attention from the surest measures for protection and prevention. Thus, in respect of smallpox, the attempt to exclude it by quarantine appears generally to have had the effect of causing the neglect of the only sure preservative, viz., the vaccination of the community. Such has been the case in many of the West India and other colonies of this country.

"It should also be borne in mind that oppressive quarantine is very apt to defeat its own purpose by its very stringency, and by making it the interest of shipmasters and others to conceal the truth, with the view of evading the annoyance and expense of a lengthened detention.

"IX.—The *sanitary and hygienic state* of *merchant shipping* is often very faulty; and there is good reason to believe that there is at all times a large amount of sickness, damaged health, and premature disablement among the merchant seamen, which might be easily prevented by simple precautionary measures.

"The *sanitary condition,* too, of most *seaport towns,* and more especially of those parts near which the shipping is lying, is generally reported to be extremely unwholesome, and calculated, if not to engender, inevitably to aggravate many of the diseases against which quarantine is directed."

The sanitary condition of our mercantile marine and of our seaport towns, in connexion with quarantine, is a subject of the highest importance, and involves considerations not merely of commercial but of national importance. It is a subject, however, which as yet has received but little attention even from those most immediately interested in it. It would be well if the Chambers of Commerce in our leading towns could be induced to bestir themselves in a matter so intimately affecting their real interests.

The Committee conclude their Report with the following Recommendations:—

"With the view of rendering the practice of quarantine, in different countries, a means of better defence against the introduction of dangerous diseases from abroad, and at the same time of improving the condition of merchant ships and the health of their crews, without any unnecessary interruption of international intercourse, we, fully recognising the great importance of an efficient sanitary supervision in all great seaports, would submit the following *recommendations* ;— Our object is to amend and utilise, not to discontinue or to abolish the existing machinery of action.

"1. As a general rule, vessels from abroad which have remained free from sickness during their voyage, and on board of which no malignant zymotic disease (chronic maladies not included) exists on arrival, and which are found on examination to be clean, and to have no putrescent or offensive cargo on board, may be at once admitted to pratique without respect to the country from whence they come.

"2. When quarantine detention is deemed necessary, whether from the actual or recent existence of a malignant disease on board, or from the foul and unwholesome state of the vessel, a careful examination should be made of her, and of all persons on board, by the quarantine medical officer, who should have the power and be charged with the responsibility of adopting such measures as each case demands.

"The healthy on board need not generally be detained, and the sooner the sick are removed out of the infected vessel to a suitable locality the better. In cases where smallpox is, or has been, on board, all unprotected persons, whether among the crew or passengers, should be vaccinated before they are permitted to disperse.

"3. Vessels arriving from abroad should be required to pump out their bilge-water, and to have their bilges thoroughly washed out before they are admitted into any crowded harbour or into docks, &c.

"The hatches also should have been occasionally kept open, and the hold aired as far as possible before arrival and admission.

"4. Before *bills of health* are given to a vessel on leaving a port, an examination should be made by a competent person to ascertain her sanitary state, and the health of her crew and all on board; and the particulars should be mentioned in the bill.

"5. Medical quarantine officers should be required to keep accurate records of all matters relating to quarantine, and to the condition and circumstances of the shipping (particularly of emigrant and immigrant vessels) arriving in and leaving their ports; and to prepare an *annual report* from the data so procured, for the information of the local authorities; and in this report, mention should be made of any epidemic visitation which may have occurred in the place during the year.

"This plan might at once be adopted in all our own colonies with advantage, and foreign Governments might be invited to follow the example.

"From the very complete statistics which appear to be kept at the great quarantine ports of Lisbon, Vigo, and Alexandria, which were readily communicated to the British consuls for the use of the Committee, it may be inferred that the Governments of Portugal, Spain, and Egypt would at once agree to give effect to such a proposal.

"Such *annual reports* would be extremely useful not only to each country and to each colony, but to all other countries, and also to the mother country of the colonies.

"It behoves Great Britain,—which is so deeply interested in all that concerns the freedom of commercial and general intercourse, as well as in maintaining in the utmost possible health a robust and efficient mercantile marine in all parts of the world, no less than in affording useful guidance to her numerous colonies in matters relating to public health,—to take the initiative in a work of this nature. Her example would be speedily followed by other countries.

"6. It would materially conduce to a thorough knowledge of the subject, and probably to the speedy adoption of a more rational and uniform practice generally, if the Government of this country instituted an investigation into the results of quarantine, and the working of quarantine establishments, in the chief ports of the south of Europe and of the Mediterranean where the system is still in greatest force, in order to ascertain the actual truth by personal observation on the spot."

Dr. Farr expressed his concurrence with the conclusions of the Report, except that he was unable to assume that "the introduction of dangerous diseases can be prevented by any quarantine regulations;" and Dr. Babington, Dr. Davy, and Mr. Wiblin appended certain suggestions on points of detail. Dr. Bryson dissented from the conclusions of the Report and the recommendations generally, adhering to the principles hitherto adopted in the practice of quarantine, and suggesting that the Government should institute an inquiry into the nature of infectious and epidemic diseases.

The Report is signed by the whole of the members of the Committee with three exceptions—Sir W. Pym, Dr. M'William, and Dr. Bryson. Sir W. Pym unhappily died before the completion of the Report; Dr. M'William was precluded from expressing any opinion upon it, from having been recently employed by the Government in a confidential inquiry into some questions connected with quarantine. The absence of Dr. M'William's opinion will be greatly regretted by all who take an interest in the subject, few men having had so wide an experience of the practical working of quarantine. Dr. Bryson's name is not attached for the reasons we have already given.

We would direct particular attention to the 5th and 6th recommendations. It would be well if they could be brought especially before the attention of the Government in the ensuing session of Parliament. There can be no question that it should be made imperative upon quarantine officers to keep records and prepare reports of the character described, if the system of quarantine is ever to become an efficient and trustworthy (not to say scientific) portion of State Medicine. We would suggest, however, that any recommendations to the Government upon this matter, should be accompanied by carefully drawn up examples, not only of the *forms* in which quarantine records should be kept, but of the mode in which the *annual reports* should be drawn up. Unless uniformity in this respect is secured, the value of both records and reports would be of little worth. Moreover, matters of this kind should not be left solely to the exercise of individual judgment.

The recommendation that Government should appoint a commission to investigate the results of the quarantine systems pursued in the chief ports of the south of Europe, is one which must commend itself to all who are wishful to get to the core of this complex subject, and we trust that the Government will not turn a deaf ear to it.

For the rest, we fully concur in the conclusions and recommendations of the Report.

An Appendix to the Report contains a singularly interesting and instructive historical sketch of quarantine legislation and practice in Great Britain, by Dr. Milroy, the Honorary Secretary to the Committee.

---

